# Facilitators and barriers to smoking cessation among minority men using the behavioral-ecological model and Behavior Change Wheel: A concept mapping study

**DOI:** 10.1371/journal.pone.0204657

**Published:** 2018-10-24

**Authors:** Nihaya Daoud, Ye Eun Jung, Ahmad Sheikh Muhammad, Ruth Weinstein, Amir Qaadny, Faten Ghattas, Mohammad Khatib, Itamar Grotto

**Affiliations:** 1 School of Public Health, Ben-Gurion University of the Negev, Beer-Sheva, Israel; 2 Division of Public Health, Ministry of Health, Jerusalem, Israel; 3 Israel Cancer Association, Nazareth, Israel; University College London, UNITED KINGDOM

## Abstract

**Background and aim:**

Universal smoking cessation strategies are not always successful for minorities, among whom smoking is highly prevalent despite high intention to quit. This study identifies facilitators for smoking cessation, as perceived by minority male smokers, that can inform a culturally appropriate national plan for smoking prevention and cessation.

**Methods:**

We conducted in 2013 a three-stage study among Arab minority male current and former smokers (ages 18–64) in Israel, among whom smoking is very high: first, a Concept Mapping (CM) study with 102 and 202 participants in the brainstorming, and sorting and rating phases respectively. Second, we assigned clusters identified in the CM study to contingency levels using the Behavioral Ecological Model (BEM). Third, we classified clusters into intervention functions and policies using the Behavior Change Wheel (BCW).

**Findings:**

The CM study revealed 58 barriers and facilitators for smoking prevention and cessation that were sorted into 11 clusters by the participants. These clusters were analogous to four BEM level contingency of smoking (social, institutional, community and individual). We classified it into two main policy categories, based on the BCW: 1- restructuring the socio-political environment of smoking through affirmative government's policies towards Arab minority in Israel, and 2-developing a culturally appropriate plan for smoking cessation in Arab local authorities including: raising awareness about tobacco hazards; enforcing anti-smoking laws; strengthening community institutional action; providing smoking cessation services; considering raising prices for tobacco products, addressing psychological sources of smoking in Arab men.

**Conclusions:**

Our study revealed barriers, facilitators and contingencies of smoking prevention and cessation with two main policy action items among the Arab minority in Israel: changing the socio-political environment of smoking, and developing a culturally appropriate smoking prevention and cessation national plan. Our study framework can inform policies and culturally appropriate interventions for smoking prevention and cessation in other minorities.

## Introduction

Tobacco use is the greatest preventable cause of premature death and chronic morbidity around the world [1]. Although a general decline in smoking prevalence is occurring globally [2], disparities in smoking between racial/ethnic groups and majority populations persist, particularly with regards to cessation [3–5]. Smoking cessation not only significantly decreases the risk of smoking-related diseases and mortality [6, 7], it is also associated with increased life expectancy [8]. Yet minority populations display significantly lower rates of successful cessation compared to majority populations [3, 9–11], despite greater intention to quit than majority groups. Such higher intention has been found among Hispanic Blacks and Hispanics in the US compared to Whites [12, 13], as well as among Bangladeshi and Pakistani minority men in the UK [11]. The general consensus in the literature is that lower cessation among minorities despite desire to quit is related to social and economic determinants [9–11, 14, 15].

Previous research has identified a number of reasons for ethnic differences in smoking cessation. First, there may be stronger cultural norms of smoking among certain ethnic groups [16]. A systemic review among Indigenous groups found that unique barriers to cessation include racism, ceremonial use of tobacco, historical factors, and specific cultural values [17]. These values include upholding sharing, kinship and reciprocity, pride, privacy, independence and self-reliance, all of which affect help-seeking behavior [17]. Among Pakistani and Bangladeshi minority group in the UK, other barriers to quitting include negative perceptions of cessation interventions, which were seen as expensive and demanding willpower, and a perception of smoking as a stress management strategy [11]. In the US, given the expense of accessing health, lack of economic resources may explain lower visits among certain ethnic groups [18]. Black and Hispanic smokers in the US are less likely to make yearly visits to a health care provider, and are thus less likely to receive counseling and cessation medications [3, 15].

Compared to the relative wealth of research on smoking cessation barriers among minorities, less information is available on cessation facilitators among these communities. Some research does show that emphasis on cultural values, particularly regarding family, seems to be effective for some communities. An intervention targeting Spanish-speaking smokers focused on Hispanic values, such as respect and upholding the family [19], had a significantly higher abstinence rate than among non-participants (27.4% vs. 20.5%) [19]. This echoes another study about the impact of second-hand smoking on children and family health and role-model pressure as psychological facilitators of cessation [20]. Among Pakistani and Bangladeshi communities in the UK, participants noted that their children, particularly daughters, put pressure on them to quit [11]. Evidence also shows that for certain communities religion also seems to act as a facilitator, particularly among Pakistani and Bangladeshi communities in the UK [11].

### The case of smoking among Arab minority men in Israel

In Israel, smoking is highly prevalent among Arab minority men compared to other population groups [21]. As of 2016, smoking prevalence among Arab minority men was almost two times greater than that of Jewish men across all age groups (43.2% vs. 27.8% respectively) [22]. The incidence of lung cancer mortality among Arab men is increasing, while that for Jewish men is decreasing [23]. The Arab minority makes up 21% of Israeli citizens [24]. They are socially and economically disadvantaged compared to their Jewish counterparts. Yet a large proportion of Arab minority men have been found to be at the pre-contemplation stage of quitting smoking (as defined by the trans-theoretical model) [25]. Arab women have the lowest smoking rates (<10%), smoking half as much as Jewish women (18.1%) [26, 27]. This low rate might be attributed to the protective effect of smoking stigma and cultural factors among minority women [21]. Although in some countries minority women make up a significantly high proportion of smokers [28, 29], current smoking demographics in Israel suggest that Arab men are a key target population for smoking cessation [21]. Cessation among Arab men can result in great direct and indirect positive effect overall for both Arab households/communities and the larger Israeli society due to their high smoking [21].

Yet, ethnic disparities in smoking in Israel seem entrenched, resisting national efforts at cessation in the overall population. Between 2009 and 2011 the government approved a national smoking prevention plan developed by the Ministry of Health (MoH) based on the World Health Organization (WHO)’s MPOWER action plan. Trained primary care clinics physicians began providing advice, referring patients to free-of-charge smoking cessation groups; and offering subsidized medications [30]. Yet, smoking cessation rates remain low (2.2%), and smoking related diseases and hazards [31] cost the Israeli national health care system close to 3.7 billion NIS annually [31]. Further, few published studies have been conducted on smoking prevention and/or cessation among ethnic/racial minority groups in Israel [21]. One study did find that only 40% of Arab men smokers report having been provided with advice to quit smoking by their primary-care physician [30]. Another study showed no significant effects from a cigarette smoking cessation intervention at one-month follow-up among Arab university students [32]. To the best of our knowledge no published results have appeared on culturally appropriate smoking cessation interventions tailored for the Arab population in Israel.

In the current study, based on the views of Arab minority men smokers in Israel, we aim to better understand facilitators and barriers to smoking cessation that can inform the design of a culturally appropriate national plan for smoking prevention and cessation.

## Methods

### Study design

**Fig 1:**
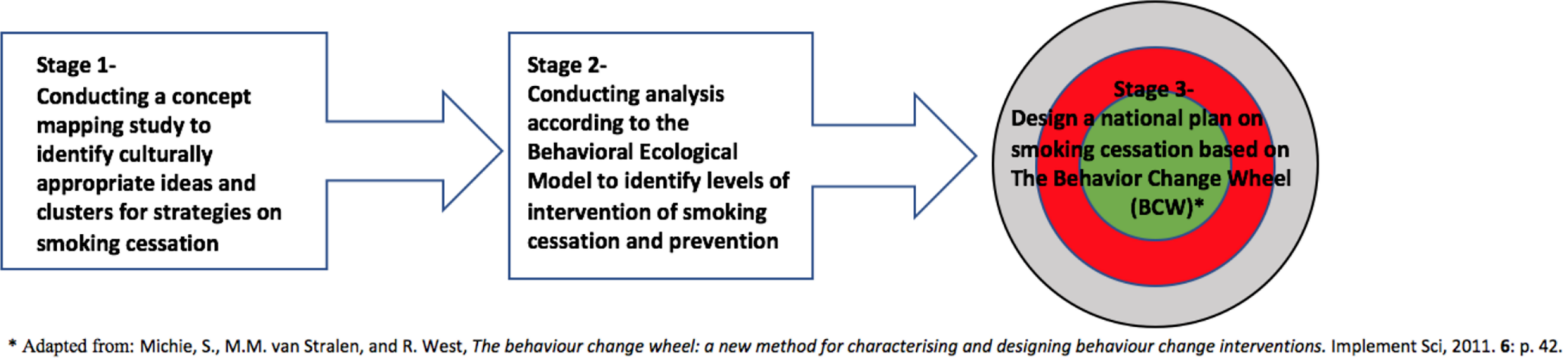
A three-stage study framework to explore facilitators to smoking cessation in minority Arab male smokers.

We conducted a three-stage study: first, a concept mapping (CM) study to elicit participants’ ideas about appropriate strategies for smoking cessation and social determinants of smoking in our study population. Second, using the Behavioral Ecological Model (BEM), we performed a thematic analysis of the CM data to better understand the contingencies of smoking (barriers, facilitators and social determinants of smoking and cessation); and third, we used the CM data and the BEM results to identify intervention functions and policy categories according to the Behavior Change Wheel (BCW) (Fig 1).

**First stage**: We used Concept Mapping (CM), a mixed and participatory research method that incorporates quantitative and qualitative data collection, and graphically present the ideas of study participants regarding a focal research question [33]. The CM has been used for planning and evaluation of health care services [34]. The CM is useful for identifying culturally appropriate smoking cessation strategies among minorities, as it enables involvement of the study participants in all stages of the study (e.g., Aboriginal-Australians) [35]. CM research involves five phases: preparation, brainstorming, sorting and rating, analyzing, and interpretation [33]. The preparation phase is designed to set up data collection and development of a focal question. In the sorting and rating phase, participants are asked to group and categorize the ideas generated in the brainstorming phase based on similarity of ideas, and to name them according to their perceptions. They are then asked to rate the ideas on a Likert format scale according to importance or relevance. In the analysis phase, Concept Systems Inc.^@^ software [36] is used to conduct multidimensional scaling and hierarchical cluster analysis in order to generate point-and-cluster maps and pattern matches that visually represent ideas and clusters of ideas. Finally, in the interpretation phase, cluster maps can be discussed with different stakeholders and drawn on for policies and interventions.

**Second stage**: We conducted thematic analysis of the data generated from the CM study using the Behavioral Ecological Model (BEM) in order to identify contingencies of tobacco control. Contingencies can be defined as factors or situations that determine smoking, responding positively or negatively to the environment (via rewards or punishments) [37]. The BEM has been used in various contexts to inform behavioral change interventions [37–40]. The BEM seeks to explain how a confluence of different agencies or smoking environments influence smoking cessation from the highest level of society (e.g., taxation of tobacco) to the individual level (e.g., physiological reactions to nicotine) [37]. The BEM assumes that these contingencies interact across all levels to influence a behavior. Hovell et al. argue that altering social/cultural systems through policy can prevent smoking and encourage cessation throughout the community [37].

**Third stage**: In conjunction with the BEM model, we refer to the Behavioral Change Wheel (BCW), a theoretically driven framework developed to typify and design interventions [41]. The wheel is made up of three layers/wheels: sources of behavior, intervention functions, and policy categories [41]. The inner layer, Sources of Behavior, includes three main components: Capability, Opportunity, and Motivation. The middle layer, Intervention Functions, identifies nine intervention types. Finally, the outer layer, Policy Categories, identifies seven types of policies. [41]. The BCW model has been applied in developing an App to increase the uptake and attendance at NHS Stop Smoking Services [42], and in designing an intervention for smoking cessation among pregnant Aboriginal women in Australia [29].

### Study population and sampling

The current study was approved by the ethics committee of the Faculty of Health Sciences at Ben-Gurion University of the Negev. The study included a convenience sample of Arab minority male smokers, as smoking is highly prevalent among Arab men compared to other population groups within Israel, including Arab women.[26]. Eligibility criteria included: 1. Arab men current or former smokers, as current smokers can inform strategies that can help them quit, and former smokers can share strategies for quitting; 2. Age 18–64 years old; this age group was targeted due to the high prevalence of smoking rates among them; smoking begins to decrease only after age 65 in this population [26, 31].

#### Stage 1: Concept mapping study

**Fig 2:**
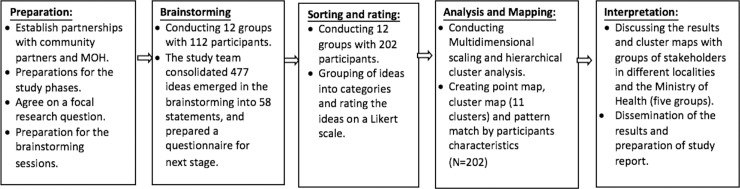
Concept mapping study phases among Arab men current and former smokers.

Data collection for the CM study took place from May to August, 2013 and included five phases, with each phase of CM building on the previous phases. Fig 2 depicts our study phases.

#### Preparation phase

In this phase we established the study team and community partners. The two main partners were: The Galilee Society (The Arab National Society for Health Research and Services) and the Israel Cancer Association branch in Nazareth, in addition to representatives from the Department of Health Promotion of the Ministry of Health (MOH). Meetings took place to discuss study design, identify data collection sites, and develop the research focal question.

#### Brainstorming phase

This phase was conducted in 11 Arab villages and towns in the north, center and south regions of Israel. Participants were recruited through the study community partners. We conducted 12 group sessions with 112 eligible participants (aged 18–64, current or former male smokers). After signing an informed consent form, participants completed a short socio-demographic questionnaire at the beginning of the group session.

The aim of brainstorming was to collect a wide range of ideas regarding the following study focal question: “*The percentage of Arab men who smoke is relatively high*. *We are interested in hearing what you believe to be the best way that could help them stop smoking*.*”* Brainstorming yielded 477 ideas generated by participants. These ideas were then consolidated by the study team into 58 statements according to similarity of content by consensus between researchers.

#### Sorting and rating phase

For the sorting activity, participants were asked to manually sort the 58 statements generated from the brainstorming phase into groups or categories. Each participant received one pile that included 58 cards, with one statement per card. Participants were instructed to sort each statement into one group only and name the group they created with logical or meaningful names. For the rating activity, participants were asked to rate their agreement with each of the statements according to relevance or effectiveness on a Likert scale, ranging from 1 (do not agree at all) to 4 (agree very much).

#### Data analysis and creating the maps

Using Concept Systems software, we conducted quantitative analyses of the data generated from the sorting and rating phase. We used multidimensional scaling and hierarchical cluster analysis to produce both a concept map and rating comparisons based on the sorting data [33]. We then used multidimensional scaling to create a point map. The map aggregates participant sorting patterns, creates x- and y- coordinates for each item, and plots them on a two-dimensional plane based on proximity [33]. The point map demonstrates a spatial arrangement of brainstormed ideas. Next we used hierarchical cluster analyses to create cluster solutions on smoking prevention and cessation that were visually displayed as boundaries around groups of points on a plot called a cluster map. These cluster solutions included a collection of brainstormed ideas on smoking prevention and cessation grouped by participants into conceptual domains. For the final cluster map solution, we began without defining the cluster’s number. This resulted in the program producing 9 cluster solutions. However, this number included some large clusters, indicating that the points were not well correlated. Therefore, we assigned more solutions until we reached 11 clusters representing an optimal and meaningful configuration of statements into clusters.

Following this, we calculated cluster ratings by averaging the rating of each item in the cluster and created a cluster rating map. Finally, we used linear correlation to compare the level of agreement on the cluster ratings of the study population subgroups using the Pearson correlation coefficient (*r)*; it ranges from 0 (no agreement between two groups) to 1 (perfect agreement between two groups). A coefficient of 0.80 or higher suggests high levels of agreement. We conducted this comparison by age group (18–25, 26–25, 36–45, 46–65), marital status (married or not married), level of education (up to high school vs. beyond high school, including college or university), employment status (yes or no), and physician advice received (yes or no).

## Results

The socio-demographic characteristics of the study participants at the brainstorming and the sorting and rating phases can be found in Table 1. In the brainstorming phase, the average age of participants was 34.2 years (SD = 14.3, range = 18–63 years). About three quarters of participants had 12 years of education, about two thirds reported full-time work, 21% reported part-time or occasional work, and the rest (about 18%) were unemployed at the time of the study. Most participants (86%) reported having a chronic illness. Most (94.6%) were current smokers, the rest past smokers. About one third were at the pre-contemplation stage for quitting smoking, 34.6% were at the contemplation stage, and the rest were at the preparation stage. However, only half of participants reported receiving physician advice to quit smoking.

**Table 1:** Characteristics of study participants in the brainstorming and sorting and rating phases.

Study variables	Participants at the brainstorming phase (N = 112)			Participants at the sorting and rating phase (N = 202)	
Age mean (SD)	34.2 (14.3)			29.9 (11.8)	
	N*	%		N*	%
**Marital status**					
Married	49	48.5		88	44.4
Not married (single, divorced, widow, separated, other)	52	51.5		110	55.6
**Education**					
Less than 12	29	33.9		29	19.3
12 years	33	38.4		80	53.3
More than 12 years	24	27.9		41	27.3
**Do you work?**					
Yes, full time	64	61.0		122	62.5
Yes, partial time	22	21.0		36	18.5
No, unemployed, pension, other	19	18.1		37	19.0
**Physician informed me that I have a chronic disease?**					
Yes	14	14.1		13	6.6
No	85	85.9		185	93.4
**Do you smoke?**					
Current smokers	106	94.6		182	91.5
Past smokers	6	5.4		17	8.5
**Intention to quit smoking**					
Yes, within the next month	40	38.5		66	37.1
Yes, within the next 6 months	36	34.6		57	32.0
No, I don't think about quitting	28	26.9		55	30.9
**Ever received physician advise to quit smoking**					
Yes	55	50.0		109	54.8
No	55	50.0		90	45.2

* Due to missing cases, the total N for different variables is different than the total N for the participants.

Characteristics of the participants at the sorting and rating phase are presented in Table 1. The mean age was close to 30. Less than half were married. Close to 53% had high school education, and most of the participants were employed full or part time at the time of the study. About 7% had at least one chronic disease. All of the participants reported current smoking, except 8.5% who reported former smoking. About one third of participants reported that they have no intention of quitting smoking. The distribution of participants across the stages of quitting smoking was almost one third at the pre-contemplation, contemplation and preparation stages. Meanwhile, almost half reported that their physician never advised them to quit.

### Point and Cluster rating maps

Brainstorming yielded 58 statements on smoking cessation and prevention (Table 2). A detailed description of the data, including the percentage of agreement for each item, is presented in the supporting information table (S1 Table).

**Table 2:** Facilitators, barriers and social determinants of smoking prevention and cessation as perceived by Arab minority men current and former smokers.

Clusters and statements	Mean score
***Cluster 1- Father’s role***	
1. First, the father must stop smoking so that his children will not smoke. The father is the role model: if he smokes, his children will smoke, and if he does not, his children will not.	3.24
2. The father is an important factor in his children smoking. He must come to an understanding with them and be their friend so he can convince them not to smoke. The father must be careful not to create a situation in which his children rebel and smoke.	3.56
3. The father must set rigid rules against smoking in the house. He must control his children so that they do not smoke.	3.28
4. The father must increase his children’s opposition to smoking, and not send them to buy cigarettes. The father has the important function of educating his children, from an early age, to hate smoking.	3.64
***Cluster 2- Raising price***s ***of tobacco-product and cigarette***	
45. Raising the price of cigarettes will not get smokers to stop smoking; they will continue to buy and smoke cigarettes.	3.03
46. Raising the price of cigarettes will cause people to stop smoking.	2.46
47. A sharp rise in the price of cigarettes, doubling the price for example, will cause people to stop smoking.	2.65
49. The price of cigarettes is increased to collect taxes from Arabs and not to encourage people to stop smoking. Increased taxation on cigarettes might bring an opposite response if the Ministry of Health does not establish a special program to get Arabs to quit smoking.	3.10
***Cluster 3- Psychological sources***	
51. The most important factor in getting someone to stop smoking is psychological; persuasion, motivation, and inner strength will bring a person to decide to stop smoking.	3.58
52. The fear of disease is important: it spurs smokers to stop out of fear for their health and for the problems their family will face when they are hospitalized.	3.35
53. Nothing will help me stop smoking. I know that every cigarette I smoke shortens my life and I feel the pain, but I’ll continue to smoke.	2.97
***Cluster 4- Government general policies towards the Arab minority in srael***	
54. Young people with a house to live in and an opportunity to work (a job) are likely to have decreased tension and reduced smoking rates.	3.17
55. A drop in stress due to economic and social problems and discrimination is likely to reduce smoking.	3.30
56. Change in government policies toward Arab society can lead to a reduction in smoking. For example, by eliminating the appointment of teachers based on favoritism.	2.71
57. Youths mature at the age of 15–16. Seeing teachers at school smoke spurs them to smoke. Teachers must stop smoking at school, and thereby serve as good role models for the students.	3.40
58. First we need to solve other problems: the low work opportunities, lack of housing for young people, high unemployment, and so forth. Then we can worry about smoking.	3.12
***Cluster 5- Enforcement of anti-smoking law in public places***	
16. The sale of cigarettes in Arab residential neighborhoods should be forbidden. Cigarettes should not be readily available, and cigarette machines should be allowed only in certain locations. This is not currently implemented.	3.24
29. Enforce the law prohibiting smoking in public places, including, for example, in schools, health-fund clinics, and local government offices. Increase inspections and imposition of fines for ofenders. This is not currently enforced.	3.50
30. Enforce the law prohibiting cigarette sales to minors under 18 years old. Require identity card checks of persons wanting to buy cigarettes. Forbid sale of single cigarettes to minors. This is not currently implemented.	3.71
31. Enforce the prohibition on smoking of cigarettes in schools. Strictly prohibit teachers from smoking on school grounds. This is not currently enforced.	3.60
33. Local authorities must take greater responsibility for preventing the sale of cigarettes in shops and for ensuring enforcement of relevant laws. Smoking should be forbidden in local authority offices. This is not currently enforced.	3.12
34. There are many water-pipe cafes in Arab villages and towns. These places should be closed, and instead facilities for young people should be established where they can spend free time. Sports clubs should be opened in every neighborhood, and a sports environment should be cultivated.	3.09
***Cluster 6- Multi-sectorial regulations and policies to prevent Smoking***	
35. The Ministry of Health should prevent the import and export of cigarettes. Selling of cigarettes should be forbidden by statute.	3.10
39. Non-smoking in the workplace should be encouraged and budgets allocated for this purpose, with employers offering a financial incentive to those who stop smoking.	3.37
41. The state must increase supervision of cigarette quality. Sometimes the filter of cigarettes can be dangerous to health.	3.32
42. Advertisements for the sale of cigarettes and tobacco products should be forbidden. Smoking by well-known personalities should not be allowed in movies and on TV programs.	3.23
43. Smoking should be forbidden while driving in a moving vehicle.	3.21
44. Smoking of water-pipe should be forbidden at work.	3.46
50. Marketing of loose tobacco products should be prohibited, and the price of cigarette substitutes should be raised to decrease their consumption.	3.02
***Cluster 7- Role of local authorities*, *institutions and Arab society leadership***	
32. All of Arab society’s institutions encourage smoking and do nothing to stop it.	2.60
36. It is important to politicize stop-smoking campaign messages as a way to implement democracy and equality and to provide Arab citizens with physical and psychological health security.	3.13
37. Arab politicians must call for a stop to smoking.	3.05
48. It is important to inform the public of the annual outlay for smoking in each Arab town and village. For example, in a certain town, NIS 28 million is spent each year for the purchase of smoking products.	3.22
***Cluster 8- Awareness raising on smoking hazards***	
5. It is important to raise awareness of the dangers of smoking and to emphasize the health, economic, and social costs of smoking.	3.63
6. It is important to raise awareness of the dangers of smoking by arranging meetings with persons who have become sick as a result of smoking. Well-known local and international personalities should be used in anti-smoking campaigns.	3.32
7. The family’s awareness of the dangers of smoking should be increased by means of lectures, films, and advanced technology. For example, a picture of the lungs damaged by smoking, SMSs via cell phones, short, emotional films, frightening photos of terminal patients.	3.44
8. It is necessary to remind parents that they are responsible for their children not becoming smokers. The mother must not give her children money to buy cigarettes.	3.39
9. Awareness of the risks involved in smoking begins in the infant years and continues through puberty and adulthood. The educational program against smoking must conform to the various age groups, and be ongoing.	3.52
10. Acquiring awareness of the dangers of smoking by self-education–through books, magazines, and the Internet–is an important means to stop smoking.	3.27
11. It is important to understand that smoking water pipes endangers health and is anti-social and a bad habit that leads to an addiction to smoking cigarettes.	3.39
12. Intensive school programs from a very early age aimed at preventing smoking should be instituted, with weekly classes and workshops to explain the dangers of smoking.	3.47
13. Health management organizations must educate and provide information on smoking, through programs, lectures, and written materials.	3.52
***Custer 9- The physician’s role in smoking prevention and cessation***	
24. Physicians who smoke in a medical clinic give a bad example, so they must quit smoking before they give advice to patients. A physician who smokes should not be allowed to give medical advice. It is forbidden for a physician to smoke in front of patients.	3.38
25. Physicians must advise patients to stop smoking, offer ways to stop, and inform them of programs, courses and nicotine substitutes.	3.48
26. Physicians must be specialists on the subject of smoking and give lectures to smokers explaining their rights with respect to stopping.	3.46
27. Physicians must inform patients of free workshops on how to stop smoking and of nicotine-replacement therapy or medication.	3.37
***Cluster 10- Local and national government actions for smoking cessation and prevention***	
14. Clubs and activities should be set up for young people to use their free time in a useful way.	3.58
15. The fieldwork necessary to prevent smoking is the responsibility of public institutions and local governments.	3.30
17. Cigarettes should not be displayed in shop windows and in the front of stores, and should be covered or hidden from view.	3.03
18. Bad taste or odor should be added to cigarettes.	2.98
19. Enticing pictures and colors should be removed from cigarette packaging and be replaced by scary pictures, which will cause people to stop smoking.	2.89
21. A hotline in Arabic to support non-smokers and persons who want to stop should be opened.	3.12
22. Advertisements and other messages should be printed explaining the political dimension of smoking; for example, that cigarette-tax revenues help support the settlements.	3.21
38. In the anti-smoking campaign, religious sources should be used: verses from the Quran, the Hadith, and religious-law rulings. Religious leaders (Imam) should note the prohibition on smoking in mosque sermons.	3.48
40. Ongoing workshops to help people quit smoking would be helpful, similar to workshops to raise awareness of the dangers of car accidents; treatment at medical clinics should be conditional on patients’ participation in these workshops.	3.24
***Cluster 11- The health care management organizations (HMO's) role in smoking cessation and prevention***	
20. It is important that HMOs help smokers switch from smoking to healthier habits, such as exercise, getting enough sleep, and other activities that enable them to cut back on smoking.	3.51
23. In every neighborhood, HMOs should set up permanent centers to wean people from smoking. The centers will provide counseling and follow-up care to aid people to stop smoking.	3.51
28. HMOs should not be required to treat smoking-induced illnesses, meaning that the patient will have to bear the cost of treatment. This will cause people to quit smoking.	2.73

S1 Fig (Supporting information) presents the point map. Each statement has an assigned *x* and *y* value, used to create a bivariate plot depicting the relative similarity of agreement on statements via their proximity in space.[34] The stress point was defined by Kane and Trochim (2007) as "the point that measures the degree to which the distances on the map are *discrepant* from the values in the input similarity matrix"[33]. Close to 95% of concept mapping projects yield a stress point ranging from 0.205 to 0.365 [33]. Lower stress point values indicate better fit. The overall fit of our data set was demonstrated by a stress point of 0.264, which indicates a good fit.

**Fig 3:**
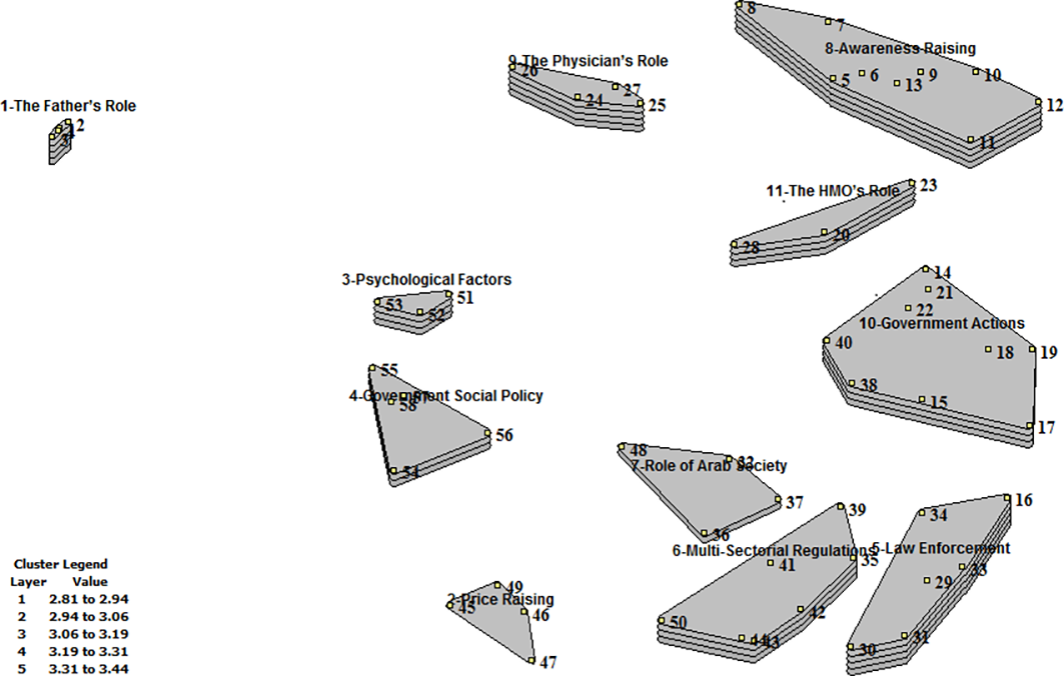
Cluster rating map of participants’ ideas on contingencies (facilitators, barriers and social determinants) of smoking prevention and cessation.

The 58 statements were sorted into 11 clusters by study participants and presented in a rating cluster map (Fig 3).

Clusters names are based on original labels the participants provided during sorting. The clusters ‘Awareness Raising’ (no. 8) and ‘Local and National Government Actions for Smoking Cessation and Prevention’ (no. 10) had the largest number of statements (9 each). These were followed by ‘Multi-Sectorial regulations’ (no. 6) and “Law Enforcement’ (no. 5), which had 6 statements each. The rest of the clusters—the Father’s role (no.1), The Physician’s role (no.9), Psychological Factors (no.3), Health Management Organization’s Role (no.11), Government Social Policies Towards Arab Society (no.4), Role of Institutions and Arab Society Leadership (no.7), and Price-Raising (no.2)—included 3–4 statements each on smoking cessation and prevention facilitators.

The layers of each cluster represent participants ratings; the more layers, the greater the average level of agreement among participants that statements within the cluster will facilitate smoking cessation and prevention.

### Clusters and statements on smoking cessation and prevention

Table 2 includes a detailed description of the clusters and statements and the mean score for each statement. The clusters included statements on smoking prevention, cessation, or both. Notably, these statements referred not just to facilitators, but also to barriers to cessation and prevention, as well as root causes (i.e., social determinants of smoking) of initiation and continuation of smoking among Arab men.

Cluster 1 focused on the father’s role as an authority and influence in Arab families. It relates to the father as a role model for his male children, emphasizing his role in educating children not to smoke, and using his authority to make them stop smoking. Most smokers in the group sessions highlighted their father’s smoking as a major factor in their own smoking initiation. Fathers can therefore be facilitators for smoking prevention and cessation if they do not smoke, do not smoke in front of their children, or quit smoking.

Cluster 2 was about raising the price of tobacco products. This cluster includes contradictory statements on raising taxes on tobacco products, based on a disparity in opinions among participants about the effectiveness of this tactic in cessation. Some participants perceived price raising or increased taxation on tobacco products as a facilitator for cessation, while others were critical of this move, stating that it is a barrier to quitting. Many participants stated that although the government collects large amounts of money (taxes) from Arab male smokers, its investment in smoking prevention and cessation programs among Arabs is minimal.

Cluster 3 focuses on the psychological factors related to smokers’ motivations to quit smoking. Some participants named lack of willpower as a barrier to quitting. Others stated that although they know about and fear the consequences of smoking, they feel that cigarettes control them. Some participants suggested that the use of negative psychology/fear in campaigns that describe scary stories about smoking hazards may convince them to stop smoking and are therefore a facilitator for cessation.

Cluster 4 relates to general government social and economic policies towards Arab society, such as allocation of funds to the education system, housing, and employment opportunities in Arab localities. Participants claimed that many of these policies are discriminatory, particularly towards young Arab men. The statements indicate that such policies increase stress among Arab men, and therefore indirectly affect their smoking. Some participants thought that changing such policies would lead to reduced stress and decreased smoking.

Cluster 5 relates to enforcement of laws governing smoking in public places. Participants in all groups agreed on the need for enforcement of anti-smoking policies in schools and public places, such as local authorities, banks, restaurants, etc. Most participants agreed that the sale of cigarettes in Arab local authorities and residential neighborhoods should be banned.

Cluster 6 looks at multi-sectoral regulations that monitor the sale and marketing of cigarettes. For example, some statements addressed regulations on the sale and advertising of cigarettes and tobacco substitutes. In addition, participants emphasized the need for regulation of workplace policies encouraging smoking cessation.

Cluster 7 looks at the role of Arab institutions and leaders in encouraging smoking cessation and providing appropriate information. Many participants agreed that information on the annual financial outlay for smoking should be made public so that people are aware of how much is being spent on smoking in each Arab village and town.

Cluster 8 statements describe specific activities to raise awareness about the health, social and economic consequences of smoking. Statements emphasized that such awareness raising should be far-reaching across age groups by utilizing various media. Statements indicate that families and education system are critical players in encouraging or preventing smoking at a young age.

Cluster 9 focuses on the role of primary-care physicians in providing advice, treatment and references for smoking prevention and cessation. There was strong consensus in favour of policies banning physicians from smoking at health-care clinics and facilities.

Cluster 10 statements relate to a variety of actions at the local and national government levels that participants felt would facilitate cessation by either addressing barriers or promoting facilitators of cessation. Participants showed a high level of consent about the idea of incorporating religious content into anti-smoking campaigns.

Cluster 11 statements relate to the role of health management organizations (HMOs) in smoking cessation. Many participants wanted HMOs to provide more services facilitating cessation, while few participants wanted HMOs to bear less burden of payment for the treatment of smoking-related illnesses in order to provide a negative incentive for smokers.

### Cluster rating scores

**Fig 4:**
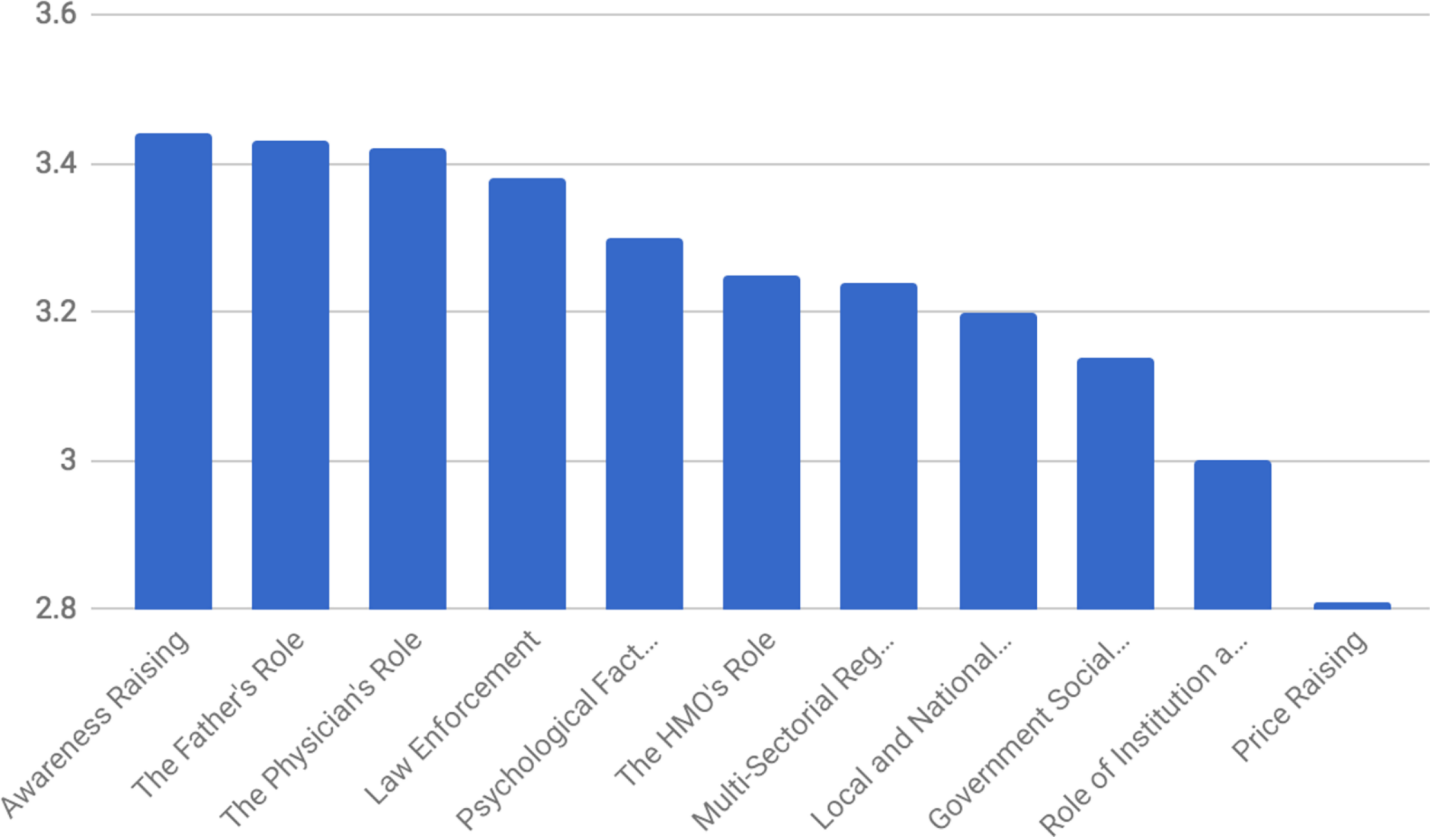
Smoking cessation cluster ratings (mean rating scores of the clusters).

Fig 4 displays the average participant level of agreement with the 11 cluster statements in bar chart form. The rating for each statement was done according to a Likert scale ranging from 1 (do not agree at all) to 4 (agree very much). The average for each statement is presented in Table 2. Fig 4 shows that the clusters of ‘awareness raising’ and ‘father’s role’ were rated highest, meaning that, on average, most participants agreed that the statements within these clusters would facilitate smoking cessation and prevention to a greater extent. Conversely, the ‘raising prices of tobacco-product and cigarette’ cluster was rated lowest, meaning that there was less agreement that statements in this cluster would facilitate cessation and prevention.

### Creating pattern matches

**Fig 5:**
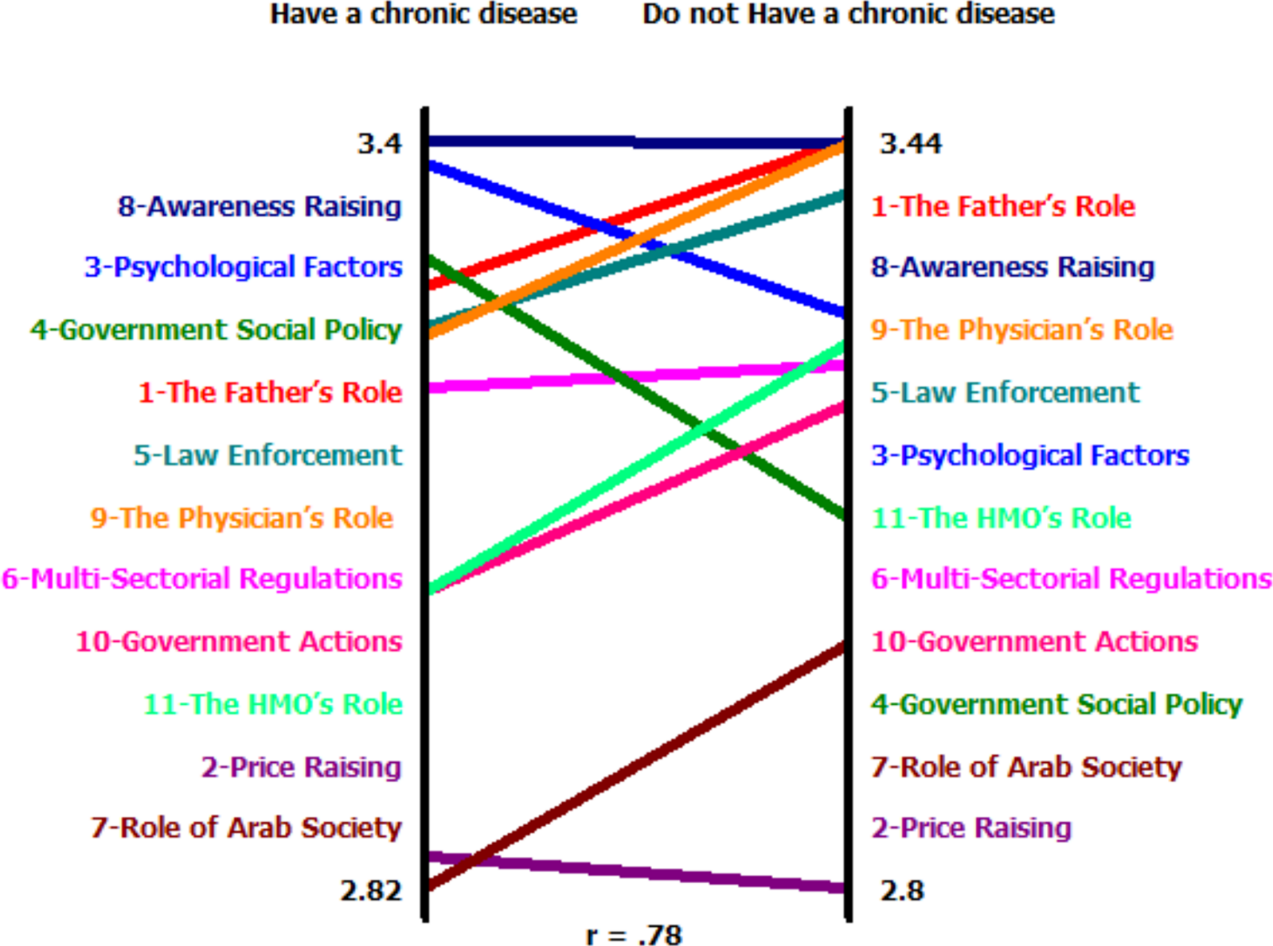
Pattern match for smoking cessation clusters among Arab men by chronic disease status.

We again used Concept Systems Inc. Software^@^ to create pattern matches. These matches compare correlations of cluster ratings according to participant characteristics. S2–S6 Fig (Supporting information) show the correlations by different socio-demographic factors. The correlations were high in all socio-demographic categories, but were lowest (r = 0.78) between those with a chronic disease and those without a chronic disease. Those with a chronic disease ranked the clusters of ‘psychological factors’ and ‘government policies’ as of greater importance than those without a chronic disease (Fig 5).

#### Interpretation phase

This phase took the form of different meetings with groups of stakeholders. This included meetings with study participants (3 groups of 42 participants), community partners at the Galilee Society and the Cancer Association branch in Nazareth (two groups of 10 participants), representatives of the Department of Health Promotion and the Public Health Division of the Ministry of health (5 participants), and one group of health promoters (15 Arab and Jewish participants) from the different districts of the Ministry of Health. All approved the results following discussion about the ideas and clusters for smoking cessation and prevention. Only slight modifications were made to the cluster labels (names) following these group discussions.

### Stage 2: Identifying BEM contingencies level

As part of our analysis, and drawing on the BEM, the researchers agreed by consensus to assign a level at which interventions could address particular contingencies identified by participants. We identified 4 levels of contingencies, which appear in Table 3: national policy−level; societal and cultural−level (including the family); local institutional−level (social and health); and individual-level contingencies. For the national policy−level contingencies, we assigned statements/clusters that shape the environment of smoking prevention and cessation. These included the following: Cluster 2 statements were largely on taxation/price-raising of tobacco products; Cluster 4 statements (except statement no. 57) were about government social policies towards the Arab minority in Israel and related to the social determinants of smoking for the Arab minority; and Cluster 6 statements were about multi-sectorial actions that can reduce smoking at the national level, including in Arab society.

**Table 3:** BEM contingencies and BCW classification of clusters on smoking cessation and prevention identified from the CM analysis among Arab minority men smokers.

Cluster statements	BEM contingencies level*	BCW Intervention Function**	BCW Policy Category***
Cluster 1- The Father's and Family Role	Social/cultural	**M**, E, P, V	**E**
Cluster 2- Price Raising	National policy	**C**, R, V	**F,** L, R, E
Cluster 3- Psychological Factors	Individual	**P**	C, U
Cluster 4- Government Policy towards the Arab minority	Socio-political level	**V, N**, M	**E, S,** L
Cluster 5- Law Enforcement (activities to enforce the laws of non-smoking in public places)	Institutional level	**R, C**, I, V, M	**L, E,** G, R, S, F
Cluster 6- Multi-Sectorial Regulations (policies and activities that can reduce smoking)	National policy; Institutional level	**R**, V, N, C, M, P	**R, L,** F, C
Cluster 7- Role of Institutions and Leadership in Arab Society	Institutional level	**E, M**, V, N, P, C	**C**, E, S
Cluster 8- Awareness Raising (activities to raise awareness on smoking hazards)	Policy level	**E, P**, V, N, C	**C, E, S **
Cluster 9- Physician's Role (The role of physicians in smoking prevention and cessation)	Institutional level	**E, P, M, T**	**S, G,** C, E, R
Cluster 10- Local and National Government Actions	National policy; Institutional level	**V, N, P, C, R, T**	**E, S, R**, L
Cluster 11- Health Care Management (HMO)’s Role in smoking cessation and prevention	National policy; Institutional level	**E, N**, V, T, C	**S, E**, R, C, G,

* Contingencies of smoking cessation include: barriers and facilitators to smoking prevention and smoking cessation and social determinants of smoking (root causes that encourage people to begin and continue smoking).

The coding key for intervention functions and policy categories was as follows

** Intervention functions: Education E, Persuasion P, Incentivization I, Coercion C, Training T, Restriction R, Environmental restructuring V, Modelling M, Enablement/resources N, Unclassifiable U

*** Policy categories: Fiscal F, Communication/marketing C, Service provision S, Legislation L, Regulation R, Guidelines G, Environmental/social planning E, Unclassifiable U

(Bold: main strategy; not bold: additional strategy)

For societal and cultural−level contingencies we assigned clusters that relate to the social and cultural processes and practices in the Arab community that create a smoking norm. Smoking is common and acceptable in all social institutions, such as schools, clinics, Mosques, local authority offices, and during social events. This level includes statements 7–9 from Cluster 8 (Awareness Raising), which discuss family responsibilities to protect children from smoking; and all the statements from Cluster 1, which refer to the father’s influence in the household.

The institutional-level contingencies included the largest number of statements (21/58). There are two types of institutions within this level: institutions run by the local municipalities, which provide services to the specific area; and institutions run by the national government, which provide social and health services. Clusters addressing local municipality institutions included statements from Cluster 5 related to law enforcement in Arab localities and institutions; statements from Cluster 7 about the role of Arab community leadership and institutions; and statements no. 14, 15 and 38 from Cluster 10 about local authorities’ actions toward smoking prevention and cessation. Statements/clusters addressing the governmental institutions included statements no. 12 and 13 from Cluster 8 that discussed the role of schools and the HMO, respectively; all statements of Cluster 9 discussing physicians’ role at primary-care clinics; and all statements of Cluster 11 about the role of HMOs in smoking prevention and cessation.

Finally, we identified few individual-level contingencies, which include motivations, feelings, such as fear, and actions related to cessation. These appeared mainly in Cluster 3 statements referring to psychosocial factors; and statements no. 10 and 11 of Cluster 8, which were connected to awareness raising.

### Stage 3: Using the Behavioral Change Wheel (BCW)

At this stage we categorized the clusters we identified in the CM analysis into the intervention functions and policy categories of the BCW, a method that can help inform the design of an intervention plan for smoking prevention and cessation in this population. Table 3 displays the 11 clusters that have been characterized as intervention functions and policy categories of the BCW. Each cluster was first coded for intervention function, followed by the policy category. This coding was done separately between 2 authors (ND and YJ) and results compared. Most of the codes on the table were categorized with full agreement/overlap. The main category/ies both researchers agreed on was/were bolded. For example, for Cluster 1, the Father’s Role, ND and YJ separately evaluated the overall statements within the cluster to infer that the main intervention function that the cluster is suggesting is Modeling, which was bolded, since the statements were related to the father’s responsibility as a role model. In addition to Modeling, we included Education, Persuasion, and Environmental restructuring (E,P,N) as part of the intervention functions for Cluster 1. The main policy category both authors agreed on for Cluster 1 was Environmental/Social Planning, which was bolded. Another example is price raising; we agreed that this mainly belonged to the intervention function of coercion (C), but we also added restrictions and environmental restructuring, when many persons will stop buying cigarettes due to high taxation. The main policy category we agreed on was fiscal measures, but we also added legislation, regulation, and environmental social planning. Based on this categorization we have identified two major policy categories: one related to the general government policies towards the Arab population in Israel (cluster 4); and one more specific to smoking policies and regulations that can be integrated within a culturally appropriate national plan for smoking cessation prevention in this population (all clusters except 4).

## Discussion

While many studies have examined barriers to smoking cessation among minorities [17], few studies have identified facilitators for quitting in these populations. We conducted a multistage analysis that includes the participatory research method of CM, the identification of contingencies based on the BEM, and the application of information gained from both to inform the design of a culturally appropriate intervention based on the BCW. Our study was conducted among Arab minority male current and past smokers in Israel, who have had high and persistent smoking rates for many decades [25, 31] compared to their Jewish counterparts [21] and to Arab women [21].

Our CM analysis revealed 11 clusters of barriers and facilitators to smoking cessation for this population. Many smoking facilitators mentioned by the men are actually rooted in multidimensional barriers to smoking prevention and cessation, and to social determinants (i.e., contingencies) of smoking [37]. While we asked mainly about facilitators to cessation, the participants emphasized the role of social, cultural, historical, and socio-political determinants in creating an environment conducive for smoking at all ages. Smoking is a collective and complex problem specifically for males in this minority. Chronic stress is an important feature inherent to this environment that stimulates and exacerbates smoking [43]. Lack of appropriate policies and interventions for smoking prevention and cessation, as well as neglect by local- and national-level governments seem to be major factors in the persistence of smoking over decades.

These results concur with a CM study conducted by Dawson et al. among Aboriginal health workers in Australia showing that efforts toward smoking cessation among minorities should be made in addition to national policies and interventions; the additional interventions need to consider contextualized cultural and social environments factors that manifest at different levels [35] including: national policy, community social institutions, the family, and the individual level. Our discussion builds on this approach and presents two main policy categories that can help restructuring the social environment and norm of smoking, based on the BCW:

### 1- Changing socio-political environment of smoking through affirmative government policies towards the Arab minority in Israel

This level applies to the environmental restructuring of BCW and relates to national policy-level contingencies towards Arabs in Israel mentioned by our study participants as facilitators for smoking cessation The participants noted that changing the Israeli government's overall policies towards the Arab citizens would reduce stress and help reduce smoking. Cluster 4 was attentive to policies affecting Israel’s Arab minority. The cluster identified longstanding government policies towards this minority as a source of stress, and statements here affirmed that smoking among Arab men is a reflection of these stressors. This finding aligns with a previous study in this population showing that smoking is related to perceived institutional and interpersonal discrimination [43]. Discrimination has previously been documented as a barrier to smoking cessation in African Americans [44] and other minorities [17]. Some studies have suggested that social exclusion and discrimination of minority men are connected to fewer opportunities for higher education and less employment, which also increases stress, decreases attentiveness to healthy behaviors and encourages smoking [17, 45], resulting in poor health [46, 47].

Arabs comprise one fifth of the population in Israel [24], but they have overall lower SES compared with their Jewish counterparts, higher poverty rates [48], lower education [49], and higher unemployment resulting from discriminatory institutional policies [50]. Health inequalities between them and the Jewish majority are related to gaps in living conditions [47]. Arab neighborhoods are characterized by high poverty and problems, including crime, violence and low collective efficacy [51, 52].

The historical socio-political context also affects the health and lifestyles of Arabs citizens in Israel. Arabs make up a native-born ethnic group that became a minority in 1948 after the establishment of the state of Israel [53]. They were under military administration for close to 18 years, which hindered their economic development [54] and political integration into Israel society [53]. They are often treated as second-class citizens [46, 55]. With few exceptions, Arabs and Jews are also enrolled in separate public education systems, with Arab schools suffering from discrimination in budgets and resources [49]. In addition, land confiscations and changing social class among Arabs have been accompanied by social and economic transitions that may have affected their lifestyles and health [56].

Participants mentioned improving housing policies and employment opportunities as important facilitators for reducing smoking, as these would reduce stress among young Arab men who struggle for both housing and jobs. They also suggested that policies should aim to reduce the socioeconomic gaps in order to reduce smoking.

### 2- Developing a culturally appropriate plan for smoking prevention and cessation with the Arab minority in Israel

This idea relates to the category of environmental social planning of the BCW, and to the intervention functions of regulations. In Clusters 6 and 10 our participants directly mentioned developing and implementing a culturally appropriate national-level plan for smoking cessation among Arabs in Israel. The National Tobacco Control Plan for smoking prevention cessation in Israel already includes an aim related to the Arab minority [41]. The plan highlighted the fact that Arabs are an at-risk population due to high smoking rates and suggested establishing a committee to examine smoking in this group and to recommend smoking strategies. However, this committee has yet to be established. Further, according to the 2017 Ministerial Report on Smoking, Israel’s MPOWER-based plan has only been partially implemented and lacks funding [31]. The current increase in smoking prevalence in Israel, specifically among Arab men [56], compared to previous years, shows that weak implementation of MPOWER has more serious implications for the Arab minority, and that culturally appropriate interventions for this group is necessary. Based on the BCW this plan should include the following interventions:

#### 2.1 Raising awareness about tobacco hazards in the Arab community

Cluster 8 calls attention to awareness raising and changing knowledge and attitudes of the Arab community regarding smoking health and economic hazards via campaigns, public lectures, school programs, and work with families, a direction that applies to communication in the BCW. Cluster 8 also emphasizes the role of schools and teachers in institutionalizing anti-smoking education from an early age. Cluster 4, regarding changing government policies towards Arabs, focuses on lack resources in schools in the Arab local authorities, thus reducing their quality and impacting teachers [49], who smoke at school and thus influence student behavior. Participants mentioned the role of families in Cluster 8, regarding awareness raising. This means that both parents need to be involved in changing attitudes towards smoking. While smoking is highly prevalent among Arab men, smoking among women in this community is exceptionally low—lower, in fact, than among Jewish women. However, smoking prevalence has been increasing in recent years, specifically among higher educated Arab women [26], which might indicate a perception of smoking as a symbol of the changing social status of Arab women [57], something which needs to be explored in future research.

#### 2.2 Enforcing anti-smoking laws in public places on the part of Arab local authorities and institutions

This level applies to 'regulations' intervention function of the BCW. It aims to change the physical or built environment of smoking through law enforcement and actions by the local municipalities, and in social and health institutions. Enforcement of anti-smoking laws have been successful in reducing smoking prevalence among low socioeconomic groups for example in Scotland [58]. In our study population, universal anti-smoking laws in Israel have not been successful in reducing smoking in Arab local authorities and institutions. Meanwhile, the role of local institutions was mentioned in 21 out of 58 statements in Clusters 5 and 7, which highlight actions for changing the physical environment of smoking. Cluster 5 was ranked high (4^th^ highest) and relates to enforcement of laws prohibiting smoking in public places, including public institutions (e.g. schools, municipal offices, community centers, weddings halls). Cluster (statements 29 and 31) focuses on schools suggesting that teachers should not smoke at schools.

As well, Clusters 4,8, 9,10 and 11 include statements on local institutions, which we highlighted in Table 2. Currently, in Arab communities in Israel, cigarettes are advertised and sold everywhere, and, although against the law, even minors can easily buy cigarettes. Participants called for reducing the presence of cigarettes in Arab neighborhoods, villages and towns by enforcing laws on advertising and marketing of tobacco products. Although local municipalities are responsible for law enforcement, due to budget shortages and low awareness few Arab authorities hire workers to carry out enforcement. Cluster 10 also suggests specific activities for prevention by limiting marketing and cigarette advertising in the Arab local authorities, and finding alternative activities, such as smoke-free clubs and cafes for adolescents, to deter them from using water pipe cafes, which are widespread in this community.

Lack of law enforcement in Arab local authorities and neighborhoods might relate to many factors. When Israel was established in 1948, many Arab villages were destroyed, and about 90% of the population was displaced. Most of the Arab population lives in separate villages and towns with close to 15% living in neighborhoods of eight mixed cities. Separation of the Arab and Jewish populations has led to segregation [43, 59], which keeps Arab localities at a lower level of services and resources compared to Jewish localities [47, 59]. Arab local authorities are constantly overwhelmed with problems, including violence and poor infrastructure [51]. While neighborhood problems and the social environment are main factors that explain ethnic inequalities in health [47], those health issues are neglected in the face of multiple other needs that also affect the social structure [51].

The specific role of Arab societal institutions emphasized in Cluster 7 criticizes these social institutions and their lack of support for smoking prevention. This cluster also emphasizes the role of Arab politicians and leaders, with participants suggesting that leaders should use political messages to speak on the rights of Arabs to live in smoke-free environment, and to highlight the economic costs of smoking. While the High Follow-Up Committee for Arab Municipalities was established to call for equal rights for the Arab population, it is not very active regarding health issues, or on smoking prevention and cessation.

#### 2.3 Providing smoking cessation services through health care services in Arab local authorities with the help of physicians

The role of physicians and health care management organizations in smoking cessation and prevention was the focus of Clusters 9 and 11. These apply to service provision and guidelines in the BCW. Previous research has shown that physicians play an important role in smoking cessation among patients [60], and that physician advice can increase readiness to quit [60]. Our participants identified this role. However, they also mentioned that physicians not always provide a positive example to quitting, as some of them smoke at the clinics instead of acting as role models. Studies show that physicians who smoke are more reluctant to provide advice on smoking cessation to patient smokers, as they do not believe in their ability to help them quit [61]. A nationwide study among Arab men in Israel showed that only 40% of male smokers have ever received primary care physician advice to quit smoking at a primary care clinic [30]. Cluster 9 highlights both barriers and facilitators of primary-care physicians’ roles. This cluster was ranked third. Some participant perceived physicians also as gatekeepers who diminish access among Arab men to cessation services, as they are less likely to inform patient smokers about free workshops and subsidized medications [30]. The participants alluded to the fact that many physicians lack proper training regarding the offer of cessation services, and said physicians should provide advice on these services and offer public lectures on smoking hazards.

The health care services role (Cluster 11) was ranked sixth. The study participants pointed to the role of health management organizations (HMOs) in training and encouraging physicians to provide advice to patient smokers. They also highlighted the role of HMOs as a facilitator not only of smoking cessation but also prevention. They mentioned that the HMOs should act at the neighborhood level to encourage healthy lifestyles, including smoke-free Arab neighborhoods. While regulations on smoking cessation from 2010 oblige HMOs to offer free cessation workshops and subsidized medications, participants noted that there aren’t enough of these in Arab communities and that smokers have to wait, sometimes more than six months, to enter a workshop, and that by then, smokers sometimes change their minds about quitting. While the 2017 Minister of Health Report on Smoking presents increased numbers of workshops on smoking cessation at each of the HMOs, there is no classification by ethnicity [26], and it is not known whether the workshops target Arab men smokers in a way that is proportional to the prevalence of smoking among them.

#### 2.4 Addressing individual-level psychological sources of smoking behavior among Arab men

While addressing psychological sources of smoking are important for behavior change based on the BCW, only few clusters included individual-level statements regarding the motivations and capabilities of smoking cessation. This low number might suggest that Arab men feel low responsibility for their smoking behavior. The presence of fatalism, denial, and machismo, which have been identified in previous research in minority men (e.g., Latino and Hispanic men in the US) [62], were also found in our study. In light of the multiple contingencies and social determinants of smoking, it seems that Arab men perceive smoking as a ‘destiny’ that cannot be changed [63]. Statement no. 53 in Cluster 3 supports this idea: “nothing will help me stop smoking and I know that every cigarette I smoke shortens my life and I feel the pain, but I’ll continue to smoke.” Fatalism might also indicate that Arab men do not feel responsible for their smoking behaviors and perceive that such behavior is shaped by the external contingencies discussed above. Therefore, they might feel less responsible for changing this situation. While fate cannot be changed, policies can affect it by changing the environment of smoking.

Other individual-level statements in Cluster 3 regarding psychological factors (statement 53) show that Arab men believe that increased motivation and willpower can facilitate smoking cessation. However, in the group’s discussions, this statement appeared to be empty and a reflection of machismo. Participants could not explain what inner strength (willpower) includes and why it isn’t used for cessation. This might indicate denial regarding smoking as an addictive behavior and the men’s inability to take steps to quit. However, participants mentioned that fear or negative psychology could increase motivation to quit. They acknowledged fearing chronic diseases (e.g., lung cancer) that are on the increase in this community [64]. Mostly, they worry for their families, as they are main breadwinners and many Arab women do not work outside the household. Chronic illness or death as a consequence of smoking among Arab males might condemn their families to poverty. Two other individual-level statements were about awareness raising and appear in Cluster 8 (no. 10 and 11). These statements match with the contemplation stage of change theory, where smokers are less prepared to take real action to quit smoking. This aligns with a recent nationwide study of this population, where many Arab men were found to be at the pre-contemplation and contemplation stages of change [25].

#### 2.5 Considering taxation policy and price-raising of tobacco products

Raising prices and taxation on tobacco products relates to the fiscal measures policy of the BCW, and to the intervention functions of restrictions and legislation [41]. Raising prices on tobacco products was mentioned in Cluster 2 statements as a facilitator to cessation as part of The National Tobacco Control Plan in Israel. While taxation has proven to be an important, cost-effective strategy for reducing the inequalities in smoking in different countries [65], there is not yet sufficient evidence on the effect of taxation in the long-term among minority groups, and this should be studied in future research [66]. Our study participants ranked price raising lowest on the list of facilitators that should be used. This might reflect their critical opinion of price-raising without the allocation of budgets for prevention and cessation in this population. They highlighted their preference for seeing the other strategies of smoking prevention and cessation being implemented first, before the government collects high taxes from this low-income population for smoking.

Taxes on cigarettes are very high in Israel compared to other countries [26]. Israel’s annual income from smoking taxes is 6–8 Billion NIS [26]. About one third of this income comes from Arab men smokers, who make up almost a third of nationwide smokers. Many low-income smokers in Israel use other tobacco products (loose tobacco) because they are cheaper, incurring lower taxes compared to packs of cigarettes. The MoH is currently seeking legislation to tax these products more highly, with little success. Some participants suggested that the government should invest some of the money from tobacco taxes into The National Tobacco Control Plan, with more investment in the Arab community that is proportionally overrepresented in smoking.

## Strengths and limitations

This is the first study we know of to use CM to identify facilitators, barriers and contingencies of smoking and cessation in minority men and to suggest interventions. The framework we developed is a study strengths and should be used in other minority groups to develop a culturally appropriate plan for smoking prevention and cessation. Using the mixed methods approach of CM for smoking cessation in a minority group is quite new [35]. In this study, CM provided a wealth of inductive data that is based on own voices of the Arab men. This increased our understanding of barriers and facilitators to cessation. The BEM analysis provided understanding on the level of contingencies of smoking and cessation. Finally, the BCW provided the framework for solutions that informed a culturally appropriate intervention. The fact that we used a convenience sample of Arab men might limit generalizability of the results. Although the socio-demographics of our sample are similar to those of participants in a random sample of Arab men smokers [25] and a national sample of Arab men [47]. Another limitation is that the study did not include Arab women. While our study rationale explains that Arab women have very low smoking, we acknowledge that their exposure to second-hand smoking and their perceptions regarding male smokers should be a focus of future research. Language was another limitation—the CM study was conducted in Arabic, however, not all research team members speak Arabic, and all the items and clusters had to be translated into English before being entered as data into the Concept Systems software. For this, we conducted translation and back-translation, which required more time and resources. In addition, there was a language barrier during consultation and interpretation sessions, since most stakeholders were not Arabic speakers. We think that speaking Arabic, the language of this minority, would be an advantage for researchers and for health-promotion professionals who work with the Arab population in Israel.

## Conclusions

In this multistage study we developed a framework that enabled us to better understand the complex problem of smoking in minority Arab men in Israel and to provide contextual solutions that can inform the design of a culturally appropriate national plan on smoking prevention and cessation. Our CM participatory research provided a wealth of ideas and revealed 11 cluster items that we classified into two main policy action items based on the BCW. These include: changing the social environmental of smoking through affirmative government policies towards the Arab minority in Israel and developing a culturally appropriate plan for smoking cessation with the Arab population including; raising awareness about tobacco hazards in the Arab population; enforcing anti-smoking laws in local institutions in the Arab local localities, particularly no smoking in public places; addressing cessation service provision by physicians and health care services in the Arab local authorities; considering taxation policy and price-raising of tobacco products; and addressing psychological sources of smoking behavior at the individual level among Arab men. Finally, implementing these action items will lead to changing the social norms of smoking. Our study framework can be used in other minority groups to inform culturally based interventions for smoking prevention and cessation.

## Supporting information

S1 Table Level of agreement (%) on each of the statements about barriers and facilitators to smoking cessation. (PDF)

S1 Fig Point map of smoking cessation facilitators, barriers and social determinants (the numbers in the map refer to the statement's number as it appears in Table 2). (TIFF)

S2 Fig Pattern match for smoking cessation clusters among Arab men by age groups. (TIFF)

S3 Fig Pattern match for smoking cessation clusters among Arab men by marital status. (TIFF)

S4 Fig Pattern match for smoking cessation clusters among Arab men by education level. (TIFF)

S5 Fig Pattern match for smoking cessation clusters among Arab men by employment status. (TIFF)

S6 Fig Pattern match for smoking cessation clusters among Arab men by physician advice received. (TIFF)

S1 Data file This is the study raw data. (MDB)
