# Supplementary material for: Facilitators and barriers to smoking cessation among minority men using the behavioral-ecological model and Behavior Change Wheel: A concept mapping study
Source: PLoS One. 2018 Oct 24;13(10):e0204657. doi: 10.1371/journal.pone.0204657 (PMC6200188; doi:10.1371/journal.pone.0204657)
Supplement: S1 Table — (PDF) [file pone.0204657.s001.pdf]

S1 Table. Level of agreement (%) on each of the statements about barriers and facilitators to smoking cessation

Participant statements	Strongly disagree N (%)	Do not agree N (%)	Agree N (%)	Strongly agree N (%)	Total N =
1. First, the father must stop smoking so that his children will not smoke. The father is the supreme ideal: if he smokes, his children will smoke, and if he does not, his children will not.	9.4 (19)	23 (11.4)	62 (30.7)	98 (48.5)	202
2. The father is an important factor on whether his children will smoke. He must come to an understanding with them and be their friend so he can convince them not to smoke. The father must be careful not to create a situation in which his children rebel and smoke. The father must identify and empathize with his children.	3 (1.5)	11 (5.4)	60 (29.7)	128 (63.4)	202
3. The father must set rigid rules against smoking in the house. He must control his children so that they do not smoke.	10 (5)	31 (15.3)	60 (29.7)	101 (50.0)	202
4. The father must increase his children's opposition to smoking, and not send them to buy cigarettes. The father has the important function of educating his children, from an early age, to hate smoking.	5 (2.5)	5 (2.5)	46 (22.9)	145 (72.1)	201
5. It is important to raise awareness of the dangers of smoking and to emphasize the health, economic, and social costs of smoking.	0 (0.0)	2 (1.0)	65 (32.5)	133 (66.5)	133
6. It is important to raise awareness of the dangers of smoking by arranging meetings with persons who have become sick as a result of smoking. Well-known local and international personalities should be used in the anti-smoking campaign.	5 (2.5)	25 (12.7)	72 (36.5)	95 (48.2)	197
7. The family's awareness of the dangers of smoking should be increased by means of lectures, films, and advanced technology. For example, a picture of the lungs damaged by smoking, SMSs via cell phones, short, emotional films, frightening photos of terminal patients.	3 (1.5)	13 (6.4)	82 (40.6)	104 (51.5)	202
8. It is necessary to remind parents that they are responsible for their children's not becoming smokers. The mother must not give her children money to buy cigarettes.	6 (3.0)	19 (9.4)	65 (32.2)	112 (55.4)	202
9. Awareness of the risks involved in smoking begins in the infant years and continues through	3 (1.5)	9 (4.5)	73 (36.7)	114 (57.3)	199

puberty and adulthood. The educational program against smoking must conform to the various age groups, and be ongoing.					
**Participant statements**	**Strongly disagree N (%)**	**Do not agree N (%)**	**Agree N (%)**	**Strongly agree N (%)**	**Total N**
10. Acquiring awareness of the dangers of smoking by self-education – through books, magazines, and the Internet – is an important means to stop smoking.	7 (3.5)	21 (10.5)	86 (43.0)	86 (43.0)	200
11. It is important to understand that smoking nargila (water pipes) endangers health and is anti-social and a bad habit that leads to an addiction to smoking cigarettes.	8 (4.0)	17 (8.5)	66 (32.8)	110 (54.7)	201
12. Intensive school programs, from a very early age, aimed at preventing smoking should be instituted, with weekly classes and workshops to explain the dangers of smoking.	4 (2.0)	10 (5.0)	77 (38.1)	111 (55.0)	202
13. The health management organizations must educate and provide information on smoking, through programs, lectures, and written materials.	(0 (0.0)	8 (4.0)	82 (40.8)	111 (55.2)	201
14. Clubs and activities should be set up for young people to use their free time in a useful way.	3 (1.5)	8 (4.0)	65 (32.3)	125 (62.2)	201
15. The fieldwork necessary to prevent smoking is the responsibility of public institutions and the local governments.	3 (1.5)	22 (11.1)	93 (46.7)	81 (40.7)	199
16. The sale of cigarettes in residential neighborhoods should be forbidden. Cigarettes should not be readily available, and cigarette machines should be allowed only in certain locations. This is not currently implemented.	10 (5.0)	35 (17.3)	63 (31.2)	94 (46.5)	202
17. Cigarettes should not be displayed in shop windows and in the front of stores, and should be covered or hidden from view.	(2 (10.4)	41 (20.3)	63 (31.2)	77 (38.1)	202
18. Bad taste or odor should be added to cigarettes.	27 (13.5)	32 (16.0)	59 (29.5)	82 (41.0)	200
19. Enticing pictures and colors should be removed from cigarette packaging and be replaced by scary pictures, which will cause people to stop smoking.	20 (10.0)	48 (23.9)	66 (32.8)	67 (33.3)	201
20. It is important that HMOs help smokers switch from smoking to healthier habits, such as exercise, getting enough sleep, and other activities that enable them to cut back on their smoking.	0 (0.0)	13 (6.5)	69 (34.3)	119 (59.2)	201

21. A hotline in Arabic to support non-smokers and persons who want to stop should be opened.	13 (6.5)	30 (14.9)	86 (42.8)	72 (35.8)	201
22. Advertisements and other literature should be printed explaining the political dimension of smoking; for example, that cigarette-tax revenues help support the settlements.	11 (5.5)	40 (19.9)	63 (30.8)	88 (43.8)	201
**Participant statements**	**Strongly disagree N (%)**	**Do not agree N (%)**	**Agree N (%)**	**Strongly agree N (%)**	**Total N**
23. In every neighborhood, HMOs should set up permanent centers to wean people from smoking. The centers will provide counseling and follow-up care to aid people to stop smoking.	3 (1.5)	9 (4.5)	75 (37.1)	115 (56.9)	202
24. Physicians who smoke in a medical clinic give a bad example, so they must quit smoking before they give advice to patients. A physician who smokes should not be allowed to give medical advice. It is forbidden for a physician to smoke in front of patients.	10 (5.0)	19 (9.5)	55 (27.5)	116 (58.0)	200
25. Physicians must advise patients to stop smoking, offer ways to stop, and inform them of programs and courses and nicotine substitutes.	2 (1.0)	8 (4.0)	83 (41.0)	54.0 (109) 109 (54.0)	202
26. Physicians must be specialists on the subject of smoking and give lectures to smokers explaining their rights with respect to stopping.	2 (1.0)	8 (4.0)	85 (42.7)	104 (52.3)	199
27. Physicians must inform patients of free workshops on how to stop smoking and of nicotine-replacement therapy or medication.	3 (1.5)	18 (8.9)	90 (44.6)	91 (45.0)	202
28. HMOs should not be required to treat smoking-induced illnesses, meaning that the patient will have to bear the cost of treatment. This will cause people to quit smoking.	43 (21.3)	40 (19.8)	56 (27.7)	63 (31.2)	202
29. Enforce the prohibition on smoking in public places law, including, for example, in schools, health-fund clinics, and local-government offices. Increase inspections and imposition of fines on lawbreakers. This is not currently enforced.	4 (2.0)	17 (8.4)	64 (31.7)	117 (57.9)	202
30. Enforce the prohibition on sale of cigarettes to minors under 18 years old law. Require identity card checks of the person wanting to buy cigarettes. Forbid sale of single cigarettes to minors. This is not currently implemented.	5 (2.5)	8 (4.0)	25 (12.4)	164 (81.2)	202
31. Enforce the prohibition on smoking of cigarettes in schools law. Strictly prohibit teachers from smoking on the school grounds. This is currently not enforced.	3 (1.5)	19 (7.9)	38 (18.8)	145 (71.8)	202

32. All of Arab society's institutions encourage smoking and do nothing to stop it.	41 (20.4)	64 (31.8)	48 (23.9)	43 (23.9)	201
33. Local authorities must take greater responsibility for preventing the sale of cigarettes in shops and for ensuring enforcement of the relevant laws. Smoking should be forbidden in local-government offices. This is not currently enforced.	12 (5.9)	39 (19.3)	74 (36.6)	77 (38.1)	202
**Participant statements**	**Strongly disagree N (%)**	**Do not agree N (%)**	**Agree N (%)**	**Strongly agree N (%)**	**Total N**
34. There are many water-pipe cafes in Arab villages and towns. These placed should be closed, and instead facilities for young people should be established where they can spend their free time should be established. Sports clubs should be opened in every neighborhood and a sports environment should be cultivated.	24 (11.9)	38 (18.8)	56 (27.7)	84 (41.6)	202
35. The Ministry of Health should prevent the import and export of cigarettes. Selling of cigarettes should be forbidden by statute.	14 (7.0)	43 (21.4)	65 (32.2)	79 (39.3)	201
36. It is important to politicize the stop-smoking campaign message, as a way to implement democracy and equality and to provide Arab citizens with physical and psychological health security.	12 (5.9)	39 (19.3)	75 (37.1)	76 (37.6)	202
37. Arab politicians must call for a stop to smoking.	14 (7.0)	37 (18.5)	81 (40.5)	68 (34.0)	200
38. In the anti-smoking campaign, religious sources should be used: verses from the Quran, the Hadith, and religious-law rulings. Religious leaders (Imam) should note the prohibition on smoking in mosque sermons.	6 (3.0)	25 (12.4)	55 (27.2)	116 (57.4)	202
39. Non-smoking in the workplace should be encouraged and budgets allocated for this purpose, with employers offering a financial incentive to those who stop smoking.	3.5 (7)	22 (10.9)	72 (35.6)	101 (50.0)	202
40. Ongoing workshops to help people quit smoking would be helpful, similar to workshops to raise awareness of the dangers of car accidents; treatment at medical clinics should be conditional on patients' participation in these workshops.	10 (5.0)	22 (10.9)	87 (43.3)	82 (40.8)	201
41. The state must increase supervision of cigarette quality. Sometimes the filter of cigarettes can be dangerous to health.	6 (3.0)	20 (10.0)	82 (41.0)	92 (46.0)	200
42. Advertisements for the sale of cigarettes and	7 (3.5)	41 (20.4)	59	94 (46.8)	201

tobacco products should be forbidden. Smoking by well-known personalities should not be allowed in movies and on TV programs.			(29.4)		
43. Smoking should be forbidden while driving in a moving vehicle.	13 (6.5)	30 (14.9)	60 (29.9)	98 (48.8)	201
**Participant statements**	**Strongly disagree N (%)**	**Do not agree N (%)**	**Agree N (%)**	**Strongly agree N (%)**	**Total N**
44. Smoking of water-pipe should be forbidden at work.	9 (4.6)	16 (8.2)	56 (28.6)	115 (58.7)	196
45. Raising the price of cigarettes will not get smokers to stop smoking; they will continue to buy and smoke cigarettes.	29 (14.6)	26 (13.1)	55 (27.8)	88 (44.4)	198
46. Raising the price of cigarettes will cause people to stop smoking.	54 (27.4)	50 (25.4)	50 (25.4)	43 (21.8)	197
47. A sharp rise in the price of cigarettes, doubling the price for example, will cause people to stop smoking.	37 (18.8)	54 (27.4)	55 (27.9)	51 (24.9)	197
48. It is important to inform the public of the annual outlay for smoking in each Arab town and village. For example, in a certain town, NIS 28 million is spent each year for the purchase of smoking products.	13 (6.5)	27 (13.5)	79 (39.5)	81 (40.5)	200
49. The price of cigarettes is increased to collect taxes from Arabs and not to encourage people to stop smoking. Increased taxation on cigarettes might bring an opposite response if the Ministry of Health does not establish a special program to get Arabs to quit smoking.	18 (8.9)	35 (17.3)	61 (30.2)	88 (43.6)	202
50. Marketing of loose tobacco products should be prohibited, and the price of cigarette substitutes should be raised to decrease their consumption.	19 (9.5)	31 (15.4)	81 (40.3)	70 (34.8)	201
51. The most important factor in getting someone to stop smoking is psychological; persuasion, motivation, and inner strength will bring a person to decide to stop smoking.	1 (0.5)	9 (4.5)	62 (31.0)	128 (64.0)	200
52. The fear of disease is important: it spurs smokers to stop out of fear for their health and for the problems their family will face when they are hospitalized.	4 (2.0)	23 (11.4)	72 (35.6)	103 (51.0)	202
53. Nothing will help me stop smoking. I know that every cigarette I smoke shortens my life and I feel the pain, but I'll continue to smoke.	13 (6.4)	46 (22.8)	79 (39.1)	64 (31.7)	202

54. Young people having a house to live in and an opportunity to work (a job) are likely to have decreased tension and reduced smoking rates.	11 (4.4)	32 (16.1)	74 (37.2)	82 (41.2)	199
55. A drop in stress due to economic and social problems and discrimination is likely to reduce smoking.	6 (3.0)	24 (12.1)	85 (42.7)	84 (42.2)	199
56. Change in government-ministry policy toward Arab society can lead to a reduction in smoking. For example, by eliminating the appointment of teachers based on favoritism.	28 (13.9)	62 (30.8)	64 (31.8)	47 (23.4)	201
57. Youths mature at the age of 15-16. Seeing teachers at school smoke spurs them to smoke. Teachers must stop smoking at school, and thereby serve as a good role model for the students to copy.	9 (4.5)	13 (6.5)	71 (35.5)	107 (53.5)	200
58. First we need to solve other problems: the low work opportunities, lack of housing for young people, high unemployment, and so forth. Then we can worry about smoking.	10 (5.0)	30 (15.0)	83 (41.5)	77 (38.5)	200
