# Supplementary figures and images for: Facilitators and barriers to smoking cessation among minority men using the behavioral-ecological model and Behavior Change Wheel: A concept mapping study
Source: PLoS One. 2018 Oct 24;13(10):e0204657. doi: 10.1371/journal.pone.0204657 (PMC6200188; doi:10.1371/journal.pone.0204657)

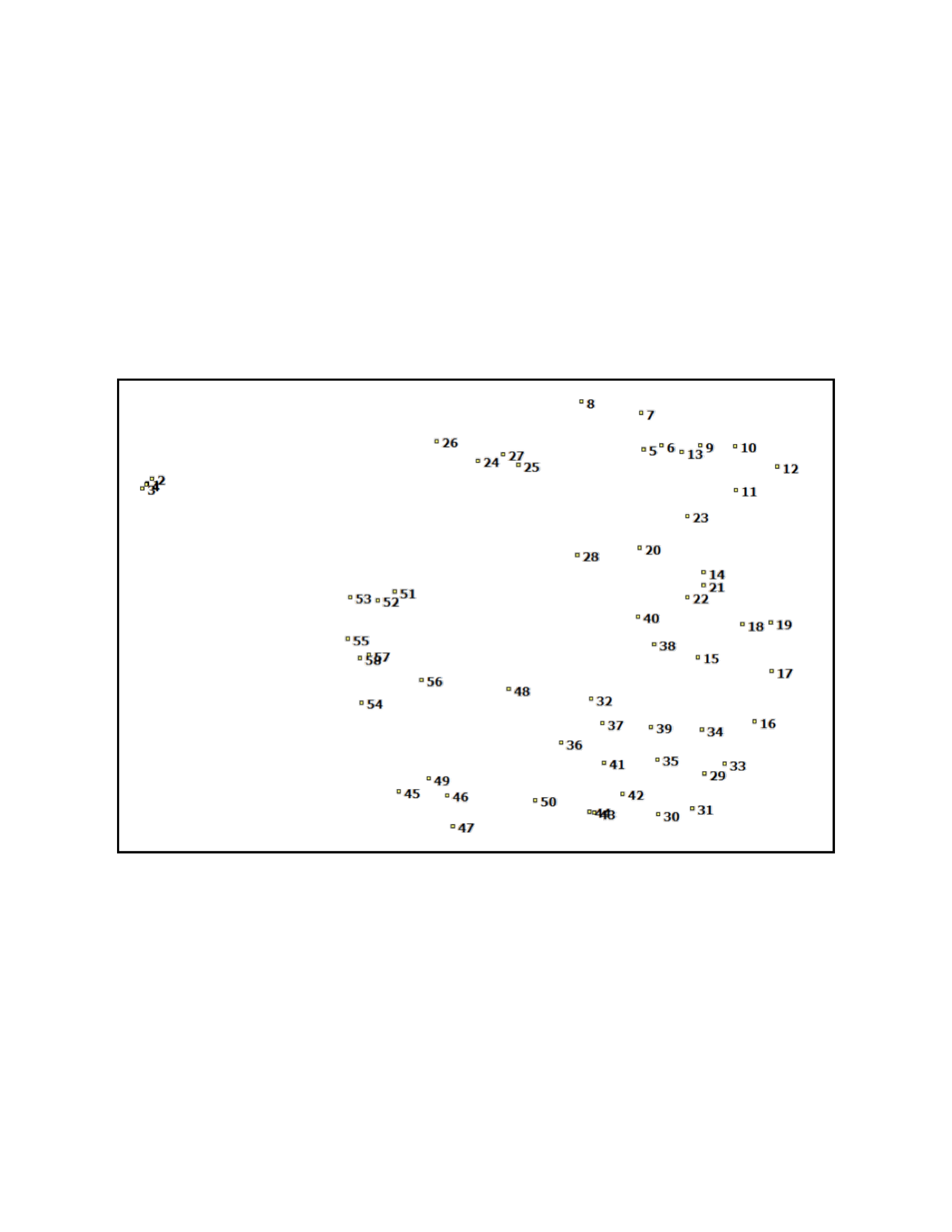

Supplement: S1 Fig — (TIFF) [file pone.0204657.s002.tiff]

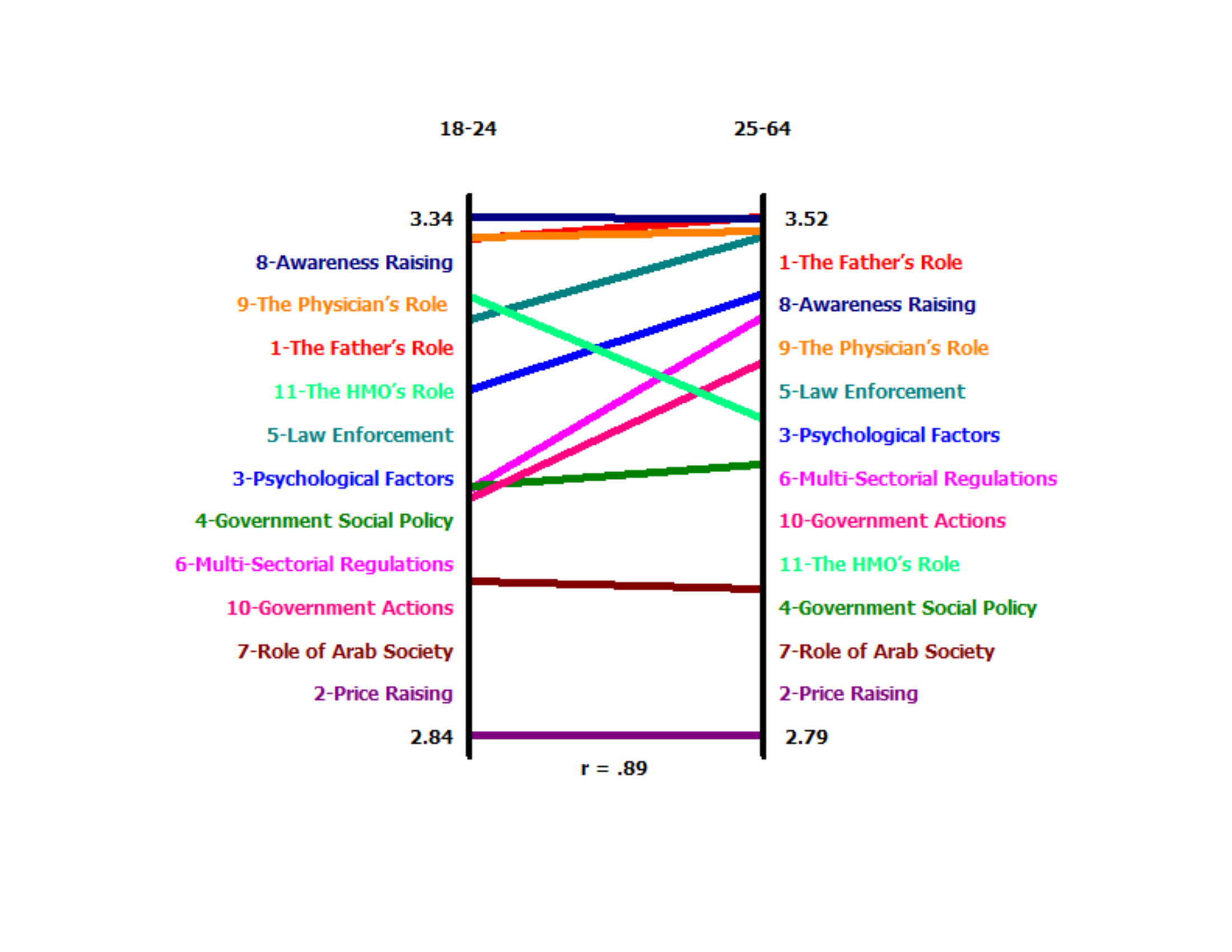

Supplement: S2 Fig — (TIFF) [file pone.0204657.s003.tiff]

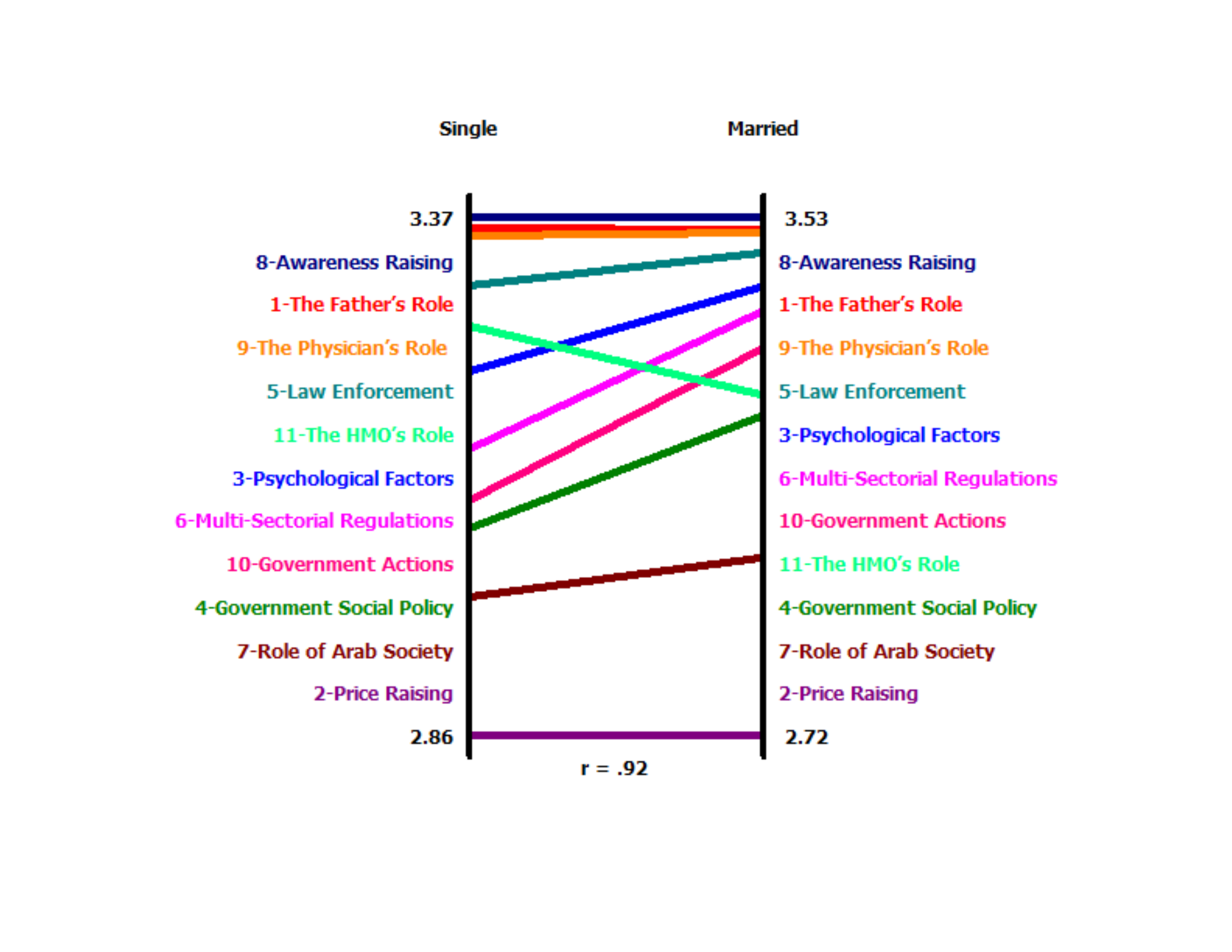

Supplement: S3 Fig — (TIFF) [file pone.0204657.s004.tiff]

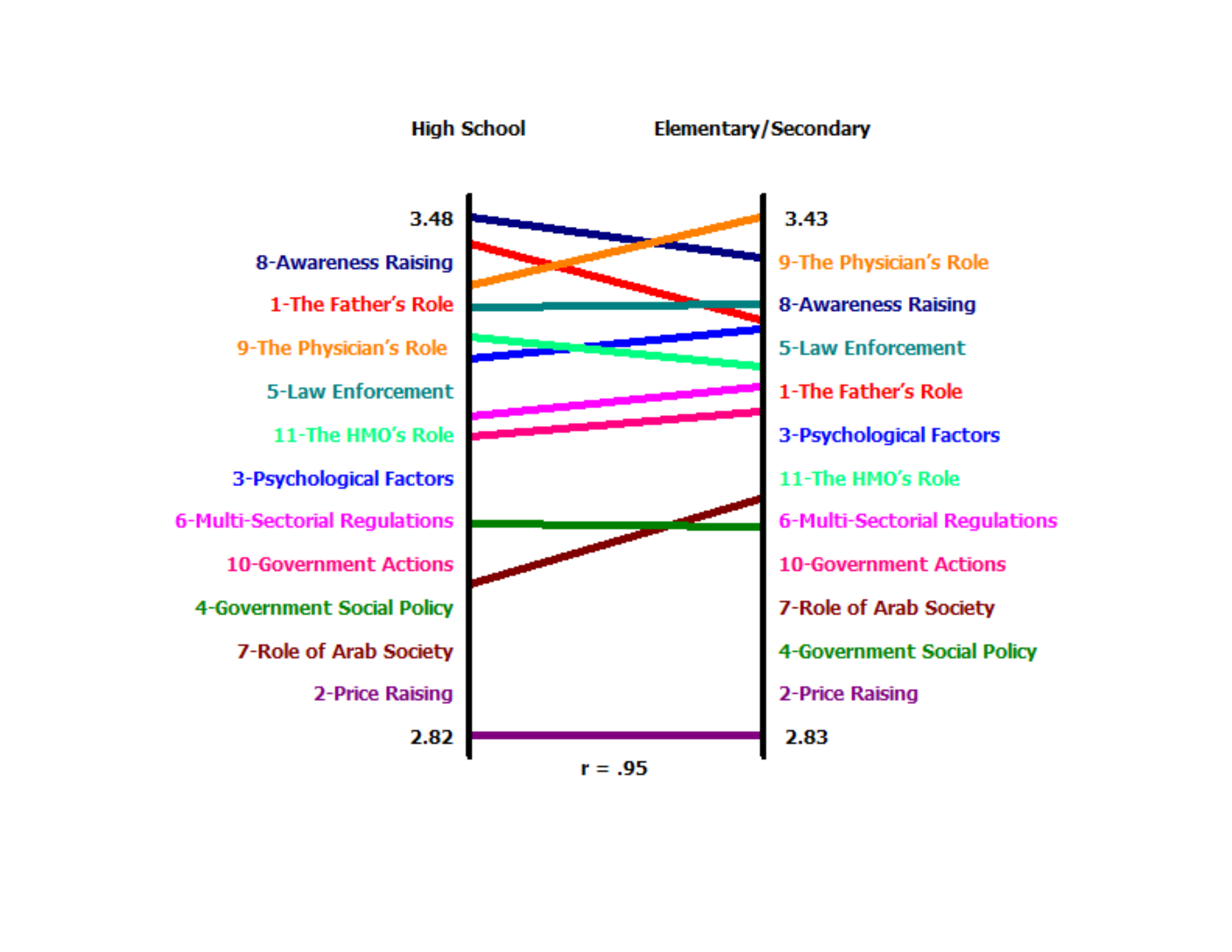

Supplement: S4 Fig — (TIFF) [file pone.0204657.s005.tiff]

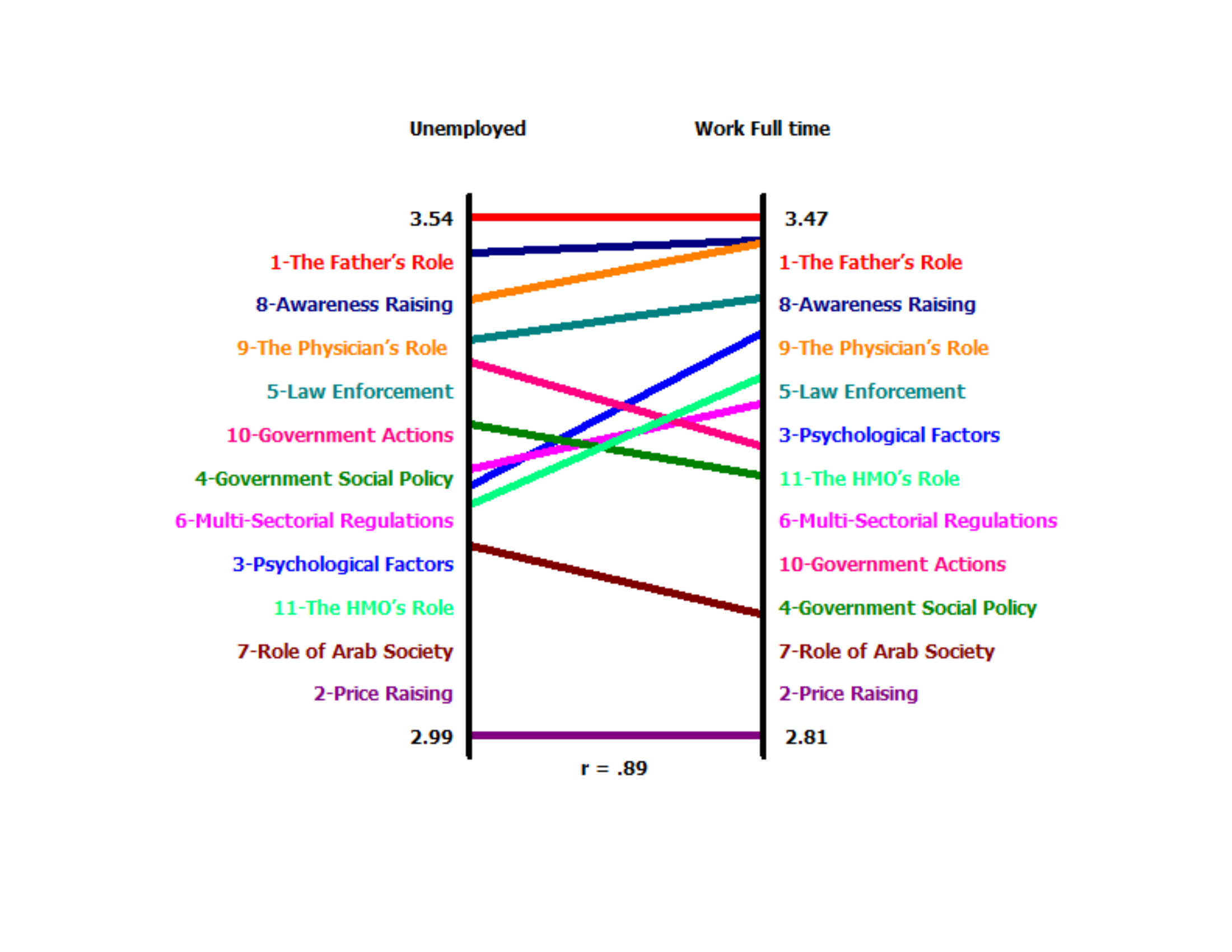

Supplement: S5 Fig — (TIFF) [file pone.0204657.s006.tiff]

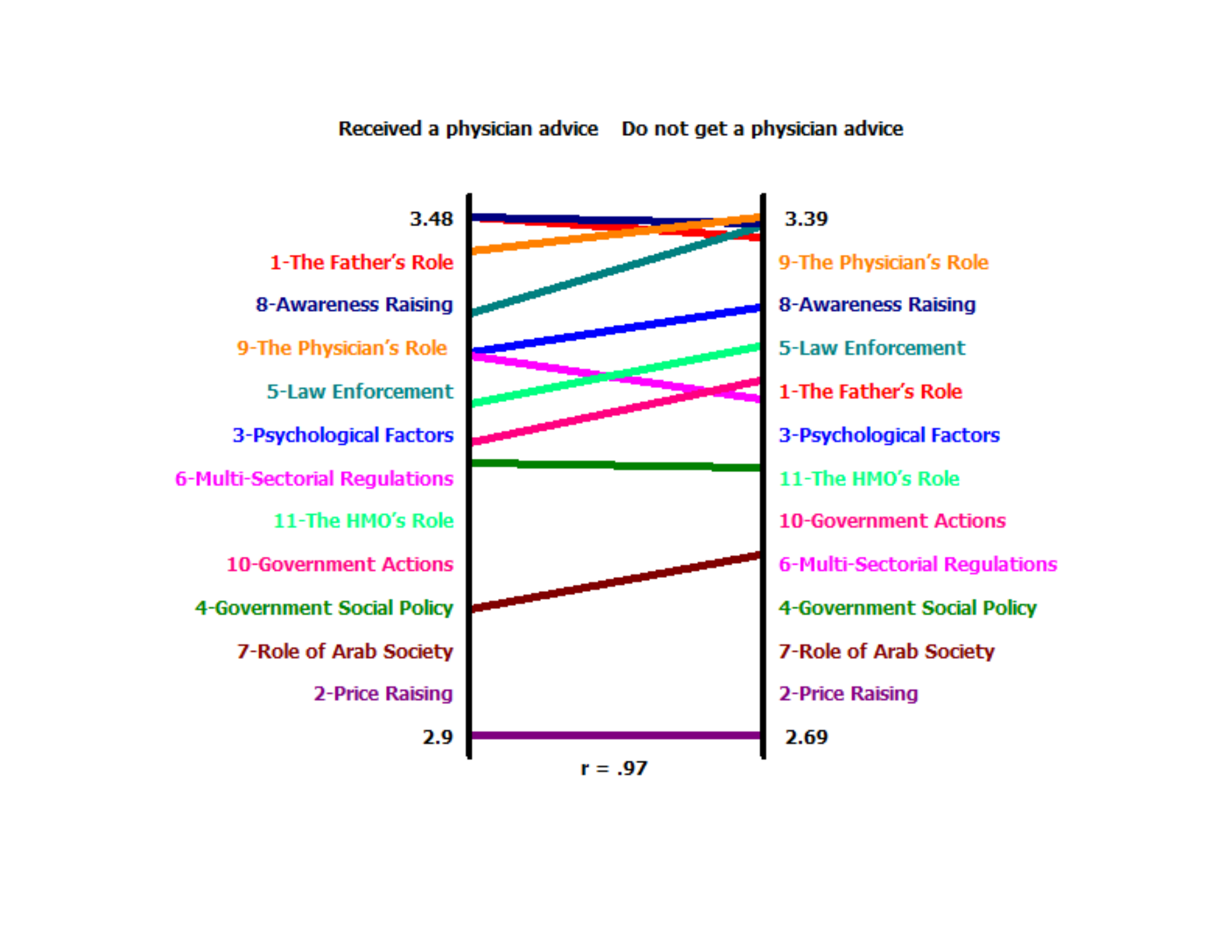

Supplement: S6 Fig — (TIFF) [file pone.0204657.s007.tiff]
